# Clinicopathologic Features of Colorectal Polyps in Shahid Beheshti University of Medical Sciences (SBMU)

**DOI:** 10.31557/APJCP.2019.20.6.1773

**Published:** 2019

**Authors:** Mahsa Ahadi, Behrang Kazemi Nejad, Zeinab Kishani Farahani, Tahmineh Mollasharifi, Elena Jamali, Hamid Mohaghegh Shalmani, Arash Dehgan, Maliheh Saberi Afsharian, Amir Sadeghi, Abolfazl Movafagh, Roxana Boran, Azadeh Rakhshan, Arsham Moradi, Mohammad Hassan Heidari, Afshin Moradi

**Affiliations:** 1 *Cancer Research Center, *; 2 *Department of pathology, *; 6 *Department of Medical Genetics,*; 8 *Department of Anatomy, School of Medicine, *; 3 *Research Center for Gastroenterology and Liver Diseases, Shahid Beheshti University of Medical Sciences, Tehran, *; 4 *Department of Pathology, Hamedan University of Medical Sciences, Hamedan, *; 5 *Department of Pathology, Azad Medical University, Mashhad, Iran, *; 7 *University of Toronto, Department of Biology, Toronto, Canada. *

**Keywords:** Colorectal polyps, adenomatous polyps, serrated polyps, non-neoplastic polyps

## Abstract

**Aim:**

This study was designed to report epidemiologic findings of polyps in Iranian patients, and predict histology of polyp regarding to demographic and colonoscopic findings.

**Background:**

Classification of colorectal polyps had been revised in the past two decades and there is a need for polyp categorization in the Iranian Health System. Patients and methods: In this retrospective study, the medical records of patients with colonoscopic diagnosis of polyp in pathology departments of SBMU affiliated teaching hospitals were reviewed. Patient’s slides evaluated and demographics findings were assessed. The anatomical location, macroscopic appearance including size and histological assessment of all polyps were recorded.

**Results:**

From total number of 1106 polyps (detected in 862 patients), adenomatous polyps (638 [57.7%]) were the most prevalent findings, followed by colon mucosal tag (184[16.6%]), hyperplastic and serrated polyps (122[11%]), inflammatory polyps (110[9.9%]), hamartomatous (21[1.9%]), and malignant lesions (13[1.2%]). Multivariate logistic regression showed age (each one year increasing age; odds ratio [OR] = 1.026, 95%confidence interval [CI] = 1.016–1.036, p < 0.0001), location of polyp (right colon; OR = 1.905, 95%CI = 1.366–2.656, p < 0.0001), and polyp size of 5-10 mm (OR = 1.662, 95%CI = 1.214–2.276, p = 0.002), and polyp size of >10 mm (OR = 2.778, 95%CI = 1.750–4.411, p< 0.0001) were independently associated with neoplastic polyps. Also, polyp size of >10 mm (OR= 2.613, 95%CI= 1.083-6.307, p=0.033), tubulovillous pattern of polyp (OR= 3.508, 95%CI= 1.666-7.387, p=0.001) and villous pattern of polyp (OR= 10.444, 95%CI= 4.211-25.905, p<0.0001) were associated with high grade dysplasia in neoplastic polyps.

**Conclusion:**

Increased age, location of polyp (right colon), increased size of polyp and villous component of polyp could classify patients in high risk groups.

## Introduction

Colorectal cancer (CRC) is the third most common cancer and cancer-related deaths in Iranian population, with an increasing trend similar to documented reports from Asia-Pacific countries (Bafandeh et al., 2008; Bokemeyer et al., 2009; Cheung et al., 2008; Moradi et al., 2009; Haggar and Boushey, 2009; Yazdizadeh et al., 2005; Khuhaprema and Srivatanakul, 2008; Zhou et al., 2017; Levin et al., 2008). Screening, early diagnosis and endoscopic removal of neoplastic polyps, as precancerous lesions, have key values in decreasing incidence of CRC (Levin et al., 2008; Siegel et al., 2012; Rex et al., 2009; Rostirolla et al., 2009). Polyps are considered neoplastic when they have dysplasia in microscopic examination. Polyps are categorized in accordance to their general properties such as gross appearance, size, number, anatomic location and histology. Histopathologic investigation could further classify them within the context of presence or absence of dysplastic features to neoplastic or non-neoplastic ones, respectively (Bacon et al., 1957; Kefeli et al., 2014; Saini et al., 2009). Over 80% of polyps found in colonoscopy are diminutive (≤5 mm) and infrequent risk of CRC, but most guidelines suggest to remove all polyps detected during colonoscopy, irrespective of their size, and send them for pathologic evaluation. 

Progression of polyps to carcinoma and adenoma-carcinoma sequence has been confirmed by several studies (Grahn and Varma, 2008; Kim and Lance, 1997; Noffsinger, 2009; Jass, 2006; Sung et al., 2005; Snover, 2011; Leggett and Whitehall, 2010). However, it is noteworthy that many detected polyps are classified to non-neoplastic lesions, such as hyperplastic polyps or colon mucosal tag in pathologic evaluation. Polypectomy of non- neoplastic lesions increases cost burdens and also its complications; so prediction of polyp histology before polypectomy could be important in the management of patients. 

Several techniques have been recommended for gastroenterologist to predict polyp histology before sending to pathologists (Kuruvilla et al., 2015; Saligram and Rastogi, 2015; Rex, 2014; Sharma et al., 2014; Coe et al., 2012). Main general hospitals of Shahid Beheshti University of Medical Sciences (SBMU), Tehran, Iran cover a large population of Tehran city and they are tertiary centers for gastrointestinal disorders. This study reviewed a large polyps data, including patients age and sex, and also polyps’ size, shape, location and histology to report epidemiologic findings of polyps in Iranian patients, and predict histology of polyp regarding to demographic and macroscopic feature of polyps in colonoscopy, such as sessile or pedunculated .

## Materials and Methods

In this study, pathology reports of colonoscopic investigation in Shohada Hospital., Modarres Hospital., Loghman Hospital., and Taleghani Hospital of SBMU between 2015 and 2017 were chosen. Pathology specimens with endoscopic diagnosis of colorectal polyps were selected. Endoscopic data were extracted from pathology requests and chart of the admitted patients. All of the slides were reviewed by seven faculty members of pathology department practicing gastrointestinal pathology in their daily routine. They made their diagnosis on their own, and then after discussing challenging cases final diagnosis were recorded based on the majority vote. 

Age, sex, number of the polyps, location of the polyps, size of the polyps, pathological grading and final pathologic diagnosis were recorded. Pathologic diagnosis of polyps were categorized as neoplastic and non- neoplastic polyps. We considered adenomatous, sessile serrated adenoma, traditional serrated adenoma. The classification of hyperplastic and serrated polyps used in this article reflects the one proposed by the World Health Organization (WHO) in 2010 (Bosman et al., 2010)

Grading of neoplastic polyps were defined as high grade and low grade neoplastic polyps (Euscher et al., 2001).

Dysplasia is classified as low grade or high grade based on the cytological and architectural features. Low-grade dysplasia is interpreted by the presence of architecturally noncomplex crypts containing nuclei that are pseudostratified, or partially stratified, and the cell nuclei reach maximally to the lower half of the cell cytoplasm. High-grade dysplasia is specified by marked pseudostratification or stratification, of neoplastic nuclei that extend up to the luminal half of the cells and commonly contain remarkable pleomorphism, increased mitotic activity, atypical mitoses, and noTable loss of polarity. 

Data are described as mean (± standard deviation) for numeric and frequency (percent) for categorical data. Data analysis was done by SPSS (ver.21) using Chi- square (exact Fisher test, if need) for categorical and independent t- test for numerical data. Adjusted multivariate logistic regression was used to predict neoplastic polyps and also high grade neoplastic polyps by demographic information and endoscopic findings. Age, sex (male, female), location of polyp (left colon, right colon), polyp count (1, >1), size (<5, 5-10, >10 mm), and polyp pattern (tubular, tubulovillus, villus, serrated polyps with dysplasia; only for predicting severity or type of dysplasia) entered to the model of multivariate logistic regression. P- value< 0.05 was considered significant. 

## Results

Within two years of the study (2015 and 2016), 1106 colorectal polyps from 862 patients comprised of 509 (59%) male and 353 (41%) female subjects were identified in four associated hospitals of SBMU. The mean age of patients was 60 years with a range of 18-90 years. The majority of the patients were in the 50-70 year age group, a small minority of them were younger than 30 years old. Most patients (79.7%) had one solitary polyp. In 19 (2.2%) patients, more than 3 polyps were detected in each patient (4 polyps in 16, 5 polyps in 2, and 6 polyps in 1 subject). Data analysis was done on 1,106 detected polyps.

 Of the 1,106 polyps, adenomatous polyps (638 [57.7%]) were the most prevalent findings (including 471 tubular adenoma, 132 tubulovillous adenoma, and 41 villous adenoma), followed by colon mucosal tag (184[16.6%]), hyperplastic and serrated polyps (122[11%]), inflammatory polyps (110[9.9%]), hamartomatous (21[1.9%]), and malignant lesions (13[1.2%]). The most common type of serrated polyps was hyperplastic ones and then SSA/P with dysplasia. The most common location for the formers was rectosigmoid and the later was transverse and ascending colon. Mucosal polyps (tags) in order of frequency were located in rectosigmoid (53.3%), transverse colon (12.5%), descending colon (12.5%), ascending colon (10.3), cecum (9.2%), and unspecified (2.2%). Table 1 shows details of pathologic diagnosis of polyps. Left colon (64%), especially rectosigmoid (52.6%), was the most common location of polyps. Most polyps had diminutive size below 5 mm (52.1%), followed by polyps with 5-10 mm (34.2%) and over 10 mm (13.7%). Of 1106 polyps, 686 (62%) were neoplastic. 

Table 2 compares demographic and polyp characteristics of neoplastic polyps vs. non- neoplastic polyps. Increased age, location in right colon, and increased size of polyp had significant association with neoplastic polyps. 

As shown in Table 3, most neoplastic polyps had low grade dysplasia (601/686) compared with high grade dysplasia (75/686). Age, male, location of left colon, size of polyps, and villus pattern had significant association with high grade dysplasia.

The final model of multivariate logistic regression on the variables showed that age (each one year increasing age; odds ratio [OR] = 1.026, 95%confidence interval [CI] = 1.016–1.036, p < 0.0001), location of polyp (right colon; OR = 1.905, 95%CI = 1.366–2.656, p < 0.0001), and polyp size of 5-10 mm (OR = 1.662, 95%CI = 1.214–2.276, p = 0.002), and polyp size of >10 mm (OR = 2.778, 95%CI = 1.750–4.411, p< 0.0001) were independently associated with neoplastic polyps (Table 4).

As shown in Table 5, polyp size of >10 mm (OR= 2.613, 95%CI= 1.083-6.307, p=0.033), tubulovillous pattern of polyp (OR= 3.508, 95%CI= 1.666-7.387, p=0.001) and villus pattern of polyp (OR= 10.444, 95%CI= 4.211-25.905, p<0.0001) were associated with high grade dyplasia in neoplastic polyps.

## Discussion

This study on a large data for resected and biopsied polyps revealed that adenomatous polyps were the most prevalent findings, followed by colon mucosal tag. Univariate study showed that increased age, location in right colon, and increased size of polyp had significant association with neoplastic polyps; moreover, age, male, location of left colon, size of polyps, and villus pattern had meaningful association with high grade dysplasia. Adjusted multivariate analysis showed that age, location in right colon and size of >5 mm were factors that independently associated with neoplastic polyps; furthermore, polyp size of >10 mm, tubulovillous and villus patterns of polyp were associated with high grade dysplasia in neoplastic polyps.

In our study, most patients were old male subjects with solitary polyp. Studies have been showed that approximately two-third of patients had solitary polyps, and advanced age is associated with detecting larger polyps (Lowenfels et al., 2011; Silva et al., 2014; Solakoğlu et al., 2014)

Of the 1,106 polyps, adenomatous polyps were the most prevalent types, followed by mucosal tags/polyps, hyperplastic and serrated polyps, inflammatory polyps, hamartomatous, and malignant lesions. Frequency of adenomatous polyps in selected Iranian studies ranged between 61% and 90% (Mirzaie et al., 2012; Darvish et al., 2013; Iravani et al., 2014; Delavari et al., 2014; Hajmanoochehri et al., 2014; Geramizadeh and Keshtkar-Jahromi, 2013), and in some other countries varies between 41% and 61% (Zhou et al., 2017; Kefeli et al., 2014; Wickramasinghe et al., 2014; Valarini et al., 2011). It seems that in some of the mentioned studies in Iran, due to over-diagnosis of non-neoplastic polyps under the name of adenomatous polyps resulted a meaningful difference in the prevalence of neoplastic polyps.

Most polyps, including adenomas, located in the left colon, especially rectosigmoid. In other Iranian reviews, 40% to 87% of polyps are located in the left colon prevailing from less than 5% to 60% (includes transverse lesions) in the right colon (Eshghi et al., 2011; Mirzaie et al., 2016; Mirzaie et al., 2012; Iravani et al., 2014; Delavari et al., 2014; Hajmanoochehri et al., 2014; Geramizadeh and Keshtkar-Jahromi, 2013). In most published data transverse colon lesions are not separated, but when considered independently, this compromises 1-15% of all colon polyps (Zhou et al., 2017; Eshghi et al., 2011; Iravani et al., 2014). There are different findings in the different part of the world; some found more adenomas in the right colon (Movafagh et al., 1996; Qumseya et al., 2012) while some detected more in the left colon (Solakoğlu et al., 2014)

In our study, most polyps had diminutive size below 5 mm, with tubular histology in the left side of colon, which is compatible with previous results (Silva et al., 2014; Sousa Andrade et al., 2008). Also, we found ninety percent of the polyps had low grade dysplasia and 10% had high grade dysplasia. Bretagne et al., (2010) conducted a study to determine the rate of high-grade dysplasia among patients with adenomatous polyps. They detected high-grade dysplasia in 32.1% of the 784 subjects. Mirzaie et al., (2012) evaluated the association between the grade of dysplasia and the location of colorectal polyps in 240 colorectal adenomatous polyps referred to the department of pathology at Rasoul-e-Akram Hospital between 2005 and 2009. They point out high grade dysplasia in 3 (6.3 %) of right-sided and 36 (18.6%) of left-sided polyps. There are two possible explanations for the diversity in the rate of high grade dysplasia prevalence; the real variation in different populations, or the natural subjectivity of the diagnosis of severity of dysplasia even with the two tier histological grading system of low and high grade dysplasia. Classification of dysplasia even with the easiest and simplest method suffers from a low coefficient of concordance (Yoon et al., 2002; Fenger et al., 1990). In the group of hyperplastic and serrated polyps there were 122(11%) polyps. We divided this group into two subgroups. The first one that accounted for 99 cases (9%) of all colon polyps, included microvesicular type hyperplastic polyps (90 cases) and goblet cell type hyperplastic polyps (9 cases). There was not any mucin-poor hyperplastic polyp in our cases. In a study at Hazrat Rasul Akram Hospital., as a pure group it accounts for 4.8% of all polyps, but in another review and as a combined class with inflammatory polyps this group constitutes 16% to 31% of all colonic polyps (Mirzaie et al., 2012). In series from other countries, this group accounts for 17% to 50% of all colonic polyps (Zhou et al., 2017; Kefeli et al., 2014; Wickramasinghe et al., 2014; Valarini et al., 2011). 

The second subgroup that comprised for 23(2%) of all colonic polyps included SSA/Ps (1 case), SSA/P with dysplasia (8 cases), traditional serrated adenoma (10 cases), conventional adenoma with serrated architecture (3 cases), and unclassifiable serrated polyps (1 case).

In Iranian studies, this group accounts for 0.3% to 2.1% of all polyps (Mirzaie et al., 2012; Iravani et al., 2014; Delavari et al., 2014; Hajmanoochehri et al., 2014; Geramizadeh and Keshtkar-Jahromi, 2013), and in other countries, this group constitutes 2% to 17% of all colonic polyps (Zhou et al., 2017; Kefeli et al., 2014; Wickramasinghe et al., 2014; Valarini et al., 2011). It seems that either these types of colonic polyps in Iran are not as common as western countries, or they remain undetected and misclassified as other polyps especially hyperplastic ones. Regarding the undeniable significance of SSA/Ps in colon carcinogenesis, proper diagnosis and stratification of these group is crucial. 

Miscellaneous polypoid lesions constituted 17% of all polyps in our series. The majority of them were mucosal tags/polyps. They usually were located in rectosigmoid with a maximum diameter of less than 5mm. As far as we aware, this is the first time that this type of polyps classified accurately in Iranian papers, and in none of the past seven large reports of colonic polyps in our country this type of polyps is indexed. There is a possibility that they classified this group under the title of hyperplastic polyps or inflammatory polyps. This is a disadvantage for referring to these works to estimate prevalence of different histologic types of polyps. In review of some of the published articles from other countries, China and Sri Lanka, they have reported 8% and 1.2% of their specimens as normal mucosa, equal to the mucosal polyps (tags) (Zhou et al., 2017; Wickramasinghe et al., 2014).

Impeccable diagnosis and classification of this group is essential for two reasons; firstly, to avoid an over diagnosis of a mostly normal polypoid mucosa as a more serious ones, and secondly, may guide the physician to consider the possibility of the presence of a significant polypoid lesion in the adjacent mucosa that better fits with clinical findings.

Inflammatory polyps accounted for 110 (9.9%) of 1106 colorectal polyps. This group represented 4.5% up to 30% of colon polyps in some series (Zhou et al., 2017; Kefeli et al., 2014; Wickramasinghe et al., 2014; Valarini et al., 2011). In most domestic works, they considered inflammatory and hyperplastic in one group and this group makes up from 5% up to 31% of colon polyps (Eshghi et al., 2011; Mirzaie et al., 2016; Mirzaie et al., 2012; Iravani et al., 2014; Delavari et al., 2014). There is a wide range in prevalence of inflammatory polyps. As the diagnosis of this group is clinically meaningful and has clinical consequences, it is advised to use strict criteria for diagnosis and to avoid using the title of inflammatory polyps as an umbrella for others such as mucosal tags or hyperplastic polyps. 

Hamartomatous polyps comprise 21(1.9%) of all cases. This group accounts for 4%-5% of colon polyps (Zhou et al., 2017; Kefeli et al., 2014; Wickramasinghe et al., 2014; Valarini et al., 2011). In most Iranian published articles, they account for 2% up to 11% of colon polyps (Eshghi et al., 2011; Mirzaie et al., 2016; Mirzaie et al., 2012; Iravani et al., 2014). The prevalence of hamartomatous polyps in our study is lower than foreign statistics but very close to the Iranian ones. The reason for this low prevalence is that the selected hospitals in our project mainly providing health services to the adult patients as the lowest age in the study was 18, and the main children hospital of our university, Mofid Hospital., is out.

Malignant polyps constituted 13 (1.2%) of all cases. In a study from Sri Lanka it accounts for 4.8% of colon polyps (Wickramasinghe et al., 2014; Haghnejad et al., 2015). In most Iranian published articles, they account for 4%-5.5% of colon polyps (Eshghi et al., 2011; Mirzaie et al., 2016; Mirzaie et al., 2012). Prevalence of malignant polyps in our cases is about of half of theirs. An explanation could be that in the current research the patients are benefited from the improved screening and early detection programs and neoplastic polyps are uncovered at earlier stages than before.

Mesenchymal polyps comprised 4(0.4%) of all cases. In several foreign country studies, they do not report any of this type of polyps (Zhou et al., 2017; Kefeli et al., 2014; Wickramasinghe et al., 2014; Valarini et al., 2011). In a study in Hazrat Rasul Akram Hospital., this group constitutes less than 2% of colon polyps (Mirzaie et al., 2012; Jamshidi et al., 2014). Our prevalence is more or less identical to the mentioned papers.

**Table 1:** Pathologic Diagnosis of Polyps (n=1106)

	No	%
A1: Microvesicular hyperplastic polyp	90	8.1
A1+A6:Microvesicular hyperplastic polyp + Sessile serrated adenoma/polyp with dysplasia	1	0.1
A1+B1:Microvesicular hyperplastic polyp+ Adenomatous polyp (tubular type)	1	0.1
A1+C1:Microvesicular hyperplastic polyp + Inflammatory Pseudo polyp	1	0.1
A2: Goblet cell hyperplastic polyp	9	0.8
A2+C2:Goblet cell hyperplastic polyp + Prolapse-Type Inflammatory polyp	1	0.1
A4: Sessile serrated adenoma/polyp	1	0.1
A5: Unclassified serrated polyp	1	0.1
A6: Sessile serrated adenoma/polyp with dysplasia	8	0.7
A7: Traditional serrated adenoma	10	0.9
A7+B4:Traditionalserrated adenoma + adenomatous polyp (tubulovillous)with high grade dysplasia	1	0.1
A8: Conventional adenoma with serrated architecture	3	0.3
B1: Adenomatous polyp (tubular type)	439	39.7
B1+A1:Adenomatous polyp (tubular type)+ Microvesicular hyperplastic polyp	3	0.3
B1+A2:Adenomatous polyp (tubular type)+Goblet cell hyperplastic polyp	1	0.1
B2: Adenomatous polyp (tubular type) with high grade dysphasia	22	2.0
B3: Adenomatous polyp (tubulovillous)	104	9.4
B4: Adenomatous polyp (tubulovillous)with high grade dysplasia	27	2.4
B4+A8:Adenomatous polyp (tubulovillous)with high grade dysplasia + Conventional adenoma with serrated architecture	1	0.1
B5: Adenomatous polyp (villous)	26	2.4
B6: Adenomatous polyp (villous)with high grade dysplasia	15	1.4
C1: Inflammatory Pseudo polyp	46	4.2
C2: Prolapse-Type Inflammatory polyp	50	4.5
C3: Inflammatory Myoglandular polyp	1	0.1
C4: Lymphoid polyp	10	0.9
C5:Inflammatory Cap polyp	3	0.3
D6: Leiomyoma of the Muscularis Mucosae	2	0.2
D9: Lipoma	2	0.2
E7: Mucosal Tag	184	16.6
E9: Fecaloid material	4	0.4
F1: Juvenile Polyps and Juvenile Polyposis	19	1.7
F1+B1:Juvenile Polyps and Juvenile Polyposis + Adenomatous polyp (tubular type)	4	0.4
F5: Juvenile Polyps and Juvenile Polyposis with low grade dysplasia	2	0.2
G1: Dysplastic-Associated lesion or Masses in inflammatory bowel Disease	1	0.1
H1: Malignant epithelial polyp	10	0.9
H2: Neuroendocrine	3	0.3
Total	1,106	100

**Table 2:** Patients’ and Polyps’ Characteristics Regarding Pathologic Types of Polyp

	Neoplastic polyps	Non- neoplastic polyps	P-value
Age	62.6±12.8	56.6±15.2	<0.0001*
Sex			0.14
Male	431 (63.8)*	245 (36.2)	
Female	255 (59.3)	175 (40.7)	
Location			<0.0001
Rectosigmoid	315 (54.4)	264 (45.6)	
Descending colon	89 (67.4)	43 (32.6)	
Transverse colon	152 (764)	47 (23.6)	
Ascending colon	78 (73.6)	28 (26.4)	
Cecum	39 (57.4)	29 (42.6)	
Unspecified	13 (59.1)	9 (40.9)	
Polyp count			0.53
1	405 (59.2)	279 (40.8)	
2	80 (65)	43 (35)	
3	18 (56.3)	14 (43.8)	
>3†	14 (73.7)	5 (26.3)	
Size			<0.0001
<5mm	316 (55.1)	258 (44.9)	
5-10mm	253 (67.3)	123 (32.7)	
>10mm	117 (75)	34 (25)	

*, Number (percent); †, In neoplastic polyps 4 polyps in 11, 5 polyps in 2, and 6 polyps in 1 subject, in non- neoplastic polyps 4 polyps in 4 subjects

**Table 3:** Patients’ and Polyps’ Characteristics Regarding Grading of Neoplastic Polyps (n= 686)

	High grade dysplasia	Low grade dysplasia	No dysplasia	P-value
Age	64.27±12.4	62.5±12.7	52.8±16.3	0.03*
Sex				0.017
Male	49 (11.4)	380 (88.2)	2 (0.5)	
Female	26 (10.2)	221 (86.7)	8 (3.1)	
Location				<0.0001
Rectosigmoid	55 (17.5)	254 (80.6)	6 (1.9)	
Descending colon	5 (5.6)	84 (94.4)	0	
Transverse colon	8 (5.3)	144 (94.7)	0	
Ascending colon	5 (6.4)	71 (91)	2 (2.6)	
Cecum	2 (5.1)	37 (94.9)	0	
Unspecified	0	11 (84.4)	2 (15.4)	
Polyp type				<0.0001
Tubular Adenoma	22 (4.7)	449 (95.3)	0	
Tubulovillus Adenoma	27 (20.5)	105 (79.5)	0	
Villus Adenoma	15 (36.6)	26 (63.4)	0	
Serrated Adenoma	1 (4)	21 (84)	3 (12)	
Polyp count				0.8
1	50 (12.3)	346 (85.5)	9 (2.2)	
2	8 (10)	72 (90)	0	
3	2 (11.1)	16 (88.9)	0	
>3‡	3 (18.2)	11 (81.8)	0	
Size				<0.0001
<5 mm	14 (4.4)	298 (94.3)	4 (1.3)	
5-10 mm	31 (12.3)	218 (86.2)	4 (1.6)	
>10 mm	30 (25.6)	85 (72.6)	2 (1.7)	

* Tukey post hoc test showed significant differences between High grade dysplasia and No dysplasia (p=0.2) and also between Low grade dysplasia and No dysplasia (p=0.045); †Number (percent); ‡ In high grade dysplasia 4 polyps in 2, and 5 polyps in 1, in low grade dysplasia 4 polyps in 9, 5 polyps in 1 and 6 polyps in 1 subject subjects

**Table 4:** Logistic Regression for Neoplastic Polyps

	Odds ratio	95% confidence interval	P-value
Age	1.026	1.016-1.036	<0.0001
Sex			
Male	Ref		
Female	1.018	0.761-1.362	0.9
Location			
Left colon	Ref		
Right colon	1.905	1.366-2.656	<0.0001
Polyp count†			
1	Ref		
>1	1.122	0.782-1.609	0.53
Size			
<5mm	Ref		
5-10mm	1.662	1.214-2.276	0.002
>10mm	2.778	1.750-4.411	<0.0001

**Table 5:** Logistic Regression for Grading of Polyps

	Odds ratio	95% confidence interval	P-value
Age	1.007	0.983-1.032	0.579
Sex			
Male	Ref		
Female	1.197	0.627-2.285	0.586
Location			
Left colon	Ref		
Right colon	0.606	0.289-1.272	0.185
Polyp count†			
1	Ref		
>1	0.867	0.401-1.874	0.717
Size			
<5 mm	Ref		
5-10 mm	2.017	0.946-4.301	0.069
>10 mm	2.613	1.083-6.307	0.033
Polyp pattern			
Tubular	Ref		
Tubulovillus	3.508	1.666-7.387	0.001
Villus	10.444	4.211-25.905	<0.0001
Serrated	0.931	0.113-7.692	0.947

Finally, 14(1.3%) of our cases were classified as mixed polyps. The most common mixed polyps were adenomatous and hyperplastic.

In two of Iranian studies, this group comprises 2.5% up to 31% of colon polyps (Mirzaie et al., 2016; Mirzaie et al., 2012; Iravani et al., 2014). Our prevalence is in between. However, marked variation in two other statistics may results from subjective nature of diagnosis of hyperplastic changes adjacent to an adenomatous polyp.

As our slides were reviewed by five experienced pathologists in the field of the gastrointestinal pathology, we have a good confidence about the precision of the diagnosis of the cases.

The main limitation to this retrospective study is lack of necessary information in some of the pathology request forms, mainly colonoscopic findings about location and gross character (sessile or pedunculated) and not using an updated and uniform descriptive terms. Another limitation to the study is that no data was available on effect of resection margin on follow up and recurrence of polyps.

Our work has led us to conclude that mucosal tags(polyps) accounts a considerable number of the polypectomy specimens, are more common in the rectosigmoid samples and most have a size of less than 5mm. In Iran, SSA/Ps are not as common as western countries, and 10 percent of adenomatous polyps displays high grade dysplasia.

In conclusion, according to our findings, increased age, location of the polyp (right colon), and larger size of the polyp were independently associated with neoplastic polyps. Moreover, polyp size of >10 mm, tubulovillous and villous were associated with high grade dysplasia in neoplastic polyps.
